# Prevalence of tracheobronchomalacia and excessive dynamic airway collapse in bronchial asthma of different severity

**DOI:** 10.1186/2049-6958-8-32

**Published:** 2013-05-14

**Authors:** Roberto W Dal Negro, Silvia Tognella, Massimo Guerriero, Claudio Micheletto

**Affiliations:** 1 Respiratory Unit, Orlandi General Hospital, Via Ospedale 2, Bussolengo, VR 37012, Italy; 2 Economics Department, Statistical Section, University of Verona, Italy; 3 Respiratory Unit, Mater Salutis Hospital, Legnago, Verona, Italy

**Keywords:** Bronchial asthma, Collapse, Excessive dynamic airway, Thyroid disorders, Tracheobronchomalacia

## Abstract

**Background:**

Tracheobronchomalacia (TBM) is a pathologic condition in which softening of tracheal and bronchial cartilage causes the dynamic narrowing of transverse or sagittal diameters of tracheobronchial lumen; an excessive dynamic airway collapse (EDAC) may also be associated, with a substantial invagination of the posterior membrane of trachebronchial tree.

The aim of this study was to assess the prevalence of both TBM and EDAC in a population of asthmatics with different degrees of disease severity compared to a reference group of subjects without any bronchial obstruction.

**Methods:**

A cohort of 202 asthmatics was investigated by means of a dynamic flexible videobronchoscopy: 74 mild persistent (MPA - age 18–68 ys; 35 males; mean FEV_1_ = 88.6% pred. ± 8.3 sd); 63 moderate (MA - age 21–71 ys; 30 males; mean FEV_1_ = 71.3% pred. ± 9.1 sd), 65 severe asthmatics (SA - age 33–70 ys; 25 males; mean FEV_1_ = 48.5% pred. ± 7.6 sd), and 62 non obstructed subjects (NO - age 18–71 ys; 38 males; mean FEV_1_ 98.6% pred. ± 2.7 sd). TBM and EDAC were classified according to FEMOS classification.

**Results:**

TBM and EDAC were observed in only 1/62 subjects (both 1.61%) of NO group, while their prevalence was 2.70% and 6.75% in MPA group; 7.93% and 19.04% in MA group; 18.46% and 69.23% in SA group, respectively. The crude prevalence of thyroid disorders in the population was 12.9%. In particular, the prevalence of thyroid disorders was significantly higher in females than in men, but 54-fold higher in females than in men in the presence of EDAC.

**Conclusions:**

1) The prevalence of both TBM and EDAC is directly related to age, gender (females), and asthma severity; 2) EDAC is much more frequent than TBM in all asthma patients; 3) both tracheal abnormalities proved to be more represented in asthmatics with thyroid disorders, and particularly in female asthmatics with EDAC.

## Background

Tracheobronchomalacia (TBM) is a pathologic condition in which softening of tracheal and bronchial cartilage causes the dynamic narrowing of transverse or sagittal diameters of tracheobronchial lumen. This condition may or may not be associated with a substantial invagination of the posterior membrane of trachebronchial tree, an entity referred to an excessive dynamic airway collapse (EDAC), which is peculiar in some patients with obstructive pulmonary disease, such as chronic bronchitis, emphysema, asthma, or can represent an isolated finding in patients during cough or forced expiration.

Tracheomalacia refers to a weakness of the trachea frequently due to a reduction and/or atrophy of the longitudinal elastic fibers of the pars membranacea or impaired cartilage integrity, such that the airway is softer and more susceptible to collapse [1,2]. This disease may arise congenitally (from disorders associated with impaired cartilage maturation or in combination with other abnormalities like tracheoesopageal fistula) or it may be acquired from prior intubation, trauma, infections, long standing extrinsic compression, or chronic inflammation [3,4]. Localized TBM is usually seen in patients with prolonged endotracheal intubation, tracheostomy, or vascular rings. The cause of diffuse TBM is often unknown, but it is frequently seen in patients with common respiratory conditions, such as chronic bronchitis.

Acquired TBM has been reported to be present in 4.5% of bronchoscopies in large general series [2]; in 12.7% of all patients undergoing bronchoscopy evaluation for respiratory disease [3], and in as many as 44% of patients undergoing bronchoscopy who have a history of chronic bronchitis [4].

It has been shown that chronic airway inflammation with diffuse panbronchiolitis might play a role in exacerbations of this disorder and the acquired form of TBM has been increasingly recognized as an important cause of chronic respiratory symptoms [5]. In TBM, central airway collapse is not closely related to airflow obstruction, and expiratory flow limitation at rest often occurs in peripheral airways without central airway collapse [6].

This pathologic airway weakening can lead to a dynamic outflow obstruction associated with symptoms (such as: dyspnea; orthopnea; intractable cough, and inability to clear secretions) which can sometimes mimic asthma worsening. Furthermore, TBM may also be associated with a substantial invagination of the posterior membrane of the tracheo-bronchial tree, an entity defined as excessive dynamic airway collapse (EDAC). [7]. To our knowledge, the incidence of TBM in patients with a reported history of asthma or Chronic Obstructive Pulmonary Disease (COPD) has not been extensively studied. However, several large studies in the general population suggest that the overall incidence of TBM is 5-10% [8,9].

However, at present, it is not generally known in which proportion patients with asthma have TBM or EDAC, and if these conditions are related to asthma severity.

The aim of this study was to evaluate the prevalence of both TBM and EDAC in a population of asthmatics of different degrees of severity compared to a reference group of subjects without any airway obstruction.

## Methods

A cohort of 202 non-smoking asthmatics was investigated: 74 mild persistent, MPA (age 18–68 years; 35 males; mean FEV_1_ = 88.6% pred. ± 8.3sd); 63 moderate asthmatics, MA (age 21–71 years; 30 males; mean FEV_1_=71.3% pred. ± 9.1sd); 65 severe asthmatics, SA (age 33–70 years; 25 males; mean FEV_1_ = 48.5% pred. ±7.6sd.

Criteria used for defining asthma in the present study were those accepted worldwide [10]. In particular, asthma diagnosis stemmed from subjects’ family and clinical data; from lung function tests (in particular, the variability of their airway obstruction; the reversibility (30′ after salbutamol 400 mcg) of their flow limitation from basal values, and/or the measurement of their bronchial response to methacholine).

All patients were already treated according to GINA guidelines [10]. All patients were in stable conditions at the time of their bronchoscopy.

Another cohort of 62 non smoking subjects without any obstructive disease, NO (age 18–71 years; 38 males; mean FEV_1_ 98.6% pred. ± 2.7sd) was also investigated from this point of view (during a bronchoscopy performed as part of an unrelated investigation) as a reference group.

None of the subjects had a systemic disease or localized conditions known as possible cause of TBM. The study was approved by the local E.C. in date January 19th, 2010 (prot. n.: 1489).

### Diagnosis

Bronchoscopic visualization of dynamic tracheal or bronchial collapse remains the gold standard for TBM diagnosis [11]. After the patients had signed the specific informed consent, all bronchoscopies were performed with topical anesthesia (lidocaine) and light sedation (midazolam 1.25 - 2.5 mg ev). The patients were studied in the supine position and had undergone standard spirometry to determine FEV_1_ the day before broncoscopy. During the examination, patients were able to breathe spontaneously and follow commands to perform deep breathing, forced exhalation, and cough maneuvers to elicit the collapsibility of the airways. The bronchoscope was introduced into the trachea, and at that point the patient was instructed to perform forced inspiratory and expiratory maneuvers with graded degrees of effort, according to a standard TBM protocol [6]. The bronchoscope was then advanced to 5 cm above the carina, and the patient was requested to perform again graded forced expiratory maneuvers. This procedure was then repeated at the entrance of the right and left main bronchi. All bronchoscopies were video recorded and reviewed after the procedure to assess the degree of airway collapse.

Obviously, the airways collapsed during forced expiration, but in some cases the airway lumen was completely obliterated as the membranous trachea had collapsed to the cartilaginous rings. Dynamic CT scan images can define the normal range of intrathoracic tracheal diameters and cross-sectional areas during forced maneuvers, and it was shown that tracheal narrowing was about 80% in patients with TBM vs 35% in healthy men [12]; Stem et al. then recommended using a cutoff of ≥ 70% narrowing on forced expiration as a diagnostic threshold for TBM [12].

### Classification

TBM and EDAC were classified according to FEMOS classification, that addresses Functional status; Extent of abnormalities notes; Morfology of the abnomarlity; Origin of the abnormality, and Severity of the disease process [7].

In the present study, only moderate (expiratory airways collapse ≥ 75%) and severe (expiratory airway collapse of 100%, such as, the airway walls make contact) TBM or EDAC were considered.

### Statistics

Quantitative variables were expressed as mean and standard deviation; categorical variables as absolute and relative frequencies. The Skewness test was used to check the normality in distribution for quantitative variables. Data were compared by t-test and Mann–Whitney test, CHI-square test, and Fisher test as appropriate.

The logistic regression model was used to estimate the odds ratio and to control the role of confounding variables; p < 0,05 was considered statistically significant. Statistical analysis was carried out by using STATA Release 12 (Stata Corp LP, Texas, USA).

## Results

Bronchoscopic visualization of trachea and main bronchi was possible in all subjects, without significant adverse events. A moderate desaturation and wheezing occurred in 42 subjects immediately after the procedure, and they were quickly recovered with inhaled salbutamol (400 μg *via* spacer) and prednisone 40 mg e.v. All patients were monitored in the 2 hours following the bronchoscopic examination and were discharged from the Lung Unit only with normal values for systemic blood pressure, heart frequency and peripheral saturation.

General characteristics of non-obstructive and of asthmatic subjects are reported in Table 1. Males were significantly prevailing in subjects of the reference group (p < 0.021); asthmatics were significantly older (p < 0.0001), and characterized by a lower mean FEV_1_ value (p < 0.0001). The overall prevalence of TBM and EDAC proved quite lower (only 1 case) in non asthmatic subjects: therefore, no statistical inference is possible for groups consisting of 1 statistical unit. When considering the whole sample of asthmatics, TBM was present in 19 cases (9.4%) and EDAC in 62 cases (30.7%), being one of these abnormalities of central airways detectable in 41.1% of a general population of asthmatics (Table 1).

**Table 1:** **General characteristics of non**-**obstructive and of asthmatic subjects**

	**Gender % m**	**Age (yr) mean ± sd**	**FEV**_**1**_**% pred. mean ± sd**	**TBM n (%)**	**EDAC n (%)**
**Non-obstructive** (n=62)	61.3	38.9±10.4	98.6±3.7	1 (1.6%)	1 (1.6%)
**Asthmatics** (n=202)	44.6	47.5±13.3	70.3±17.7	19 (9.4%)	62 (30.7%)
*p*	0.021	0.0001	0.0001	-	-

General characteristics of asthmatic subjects are reported in Table 2 by asthma severity. Females were significantly more represented in severe asthmatics (p < 0.01).

Moreover, TBM was found in 2 (2.70%) and EDAC in 5 cases (6.75%) of mild persistent asthma (MPA), while TBM was found in 5 (7.93%) and EDAC in 12 cases (19.04%) of moderate persistent asthma (MA). Finally, TBM was found in 12 (18.46%) and EDAC in 45 cases (69.23%) of severe persistent asthma (SA). Both the TBM and the EDAC prevalence, as well as their ratio, are directly and significantly related (both p < 0.0001) to asthma severity according to an exponential function, particularly in the case of EDAC (Table 2).

**Table 2:** General characteristics of asthma subjects by their asthma severity, and the corresponding distribution of TBM and EDAC

	**Gender % m**	**Age (yr) mean±sd**	**FEV**_**1**_**% pred. mean±sd**	**TBM n (%)**	**EDAC n (%)**
Mild Persistent Asthma (n = 74)	47.3	40.8 ± 14.1	88.6 ± 5.6	2 (2.70%)	5 (6.75%)
Moderate Asthma (n = 63)	47.6	49.3 ± 12.2	71.4 ± 5.4	5 (7.93%)	12 (19.04%)
Severe Asthma (n = 65)	38.5	53.4 ± 9.9	48.5 ± 6.8	12 (18.46)	45 (69.23%)
p*	0.01 (Mild-moderate vs severe)	0.0001	0.0001	0.0001	0.0001

*anova.

When compared to that of asthmatics without any tracheal abnormalities, the mean age in those with TBM or EDAC was higher (56.4 ± 10.2sd yr vs 44.6 ±13.0sd yr, p < 0.0001, and 51.8 ± 12.5sd yr vs 43.5 ± 12.8 sd yr, p < 0.001, respectively).

The crude prevalence of thyroid disorders in the population was 12.9% (34/264): in particular: thyroidectomy 52.9% (18/34); thyroiditis 26.5% (9/34); goiter 14.8% (5/34); malignancy 2.9% (1/34); other 2.9% (1/34) (Table 3). When the prevalence of thyroid disorders was investigated by gender, the OR calculated was = 8.77 (95% CI = 2.94-35.13 (by the Fischer Exact Confidence method), such as the probability for the presence of thyroid disturbances was 5-fold higher in females than in males. Moreover, when the prevalence of both EDAC and thyroid disorders was compared by gender, the OR calculated was = 54.76 (95% CI = 17.48-213.06), such as, the probability for the presence of thyroid disturbances was 54-fold higher in females than in males with EDAC.

**Table 3:** Gender dependency of TBM or EDAC and thyroid disorders

**Gender**	**n**	**%**		**n**	**%**
males	4/128	3.1	TBM	4/4	100
			EDAC	0/4	0.0
females	30/136	22.1	TBM	7/30	23.3
			EDAC	23/30	76.7

## Discussion

During normal expiration, there is a physiologic reduction in all dimensions of intrathoracic airways; when changes occurring during forced inspiration and expiration are compared by a dynamic CT, a mean 35% decrease in the cross-sectional area of the trachea has been described [12]. Thus, a certain degree of dynamic airway collapse, characterized by invagination of the posterior membrane and narrowing of the cross-sectional area of the tracheobronchial tree, represents a physiologic event. However, this phenomenon is exaggerated in some patients suffering from obstructive airway disease, or it represents an isolated finding in some patients during cough and forced expiration.

TBM is an increasingly recognized abnormality of the central airways and generally results from weakness of the tracheal or mainstem bronchial walls caused by either softening of the supporting cartilaginous rings, or redundancy of the connective tissue of the posterior membrane due to a reduction in size and number of elastic fibers, or both. TBM has been called tracheobronchial collapse, expiratory tracheobronchial collapse, expiratory tracheobronchial stenosis, and tracheobronchial dyskinesia. These various terms have not only provided an incomplete understanding of the pathophysiology of malacia and EDAC, but have also contributed to the existing confusion concerning these obstructive airway disorders and to maintain some of the uncertainty on the optimal patients’ management. Most published studies are case series and retrospective descriptions; in addition, few investigators regard malacia and EDAC as separate entities, often using the two words interchangeably.

The clinical impact of these abnormalities is quite high. The pathologic weakening of the airway can produce dynamic outflow obstruction and severe symptoms, such as: cough, dyspnea, and wheezing resistant to corticosteroids and inhaled bronchodilators, stridor, recurrent bronchitis or pneumonia, atelectasis, difficult secretions clearing, and respiratory insufficiency. Moreover, air trapping was also observed with a higher frequency and greater severity in patients with tracheobronchomalacia when compared to patients of similar ages without TBM [13], and up to 7% of patients with severe diffuse TBM may require mechanical ventilation due to respiratory failure. [14]. Current treatments include techniques that splint the central airways, such as continuous positive pressure ventilation [15], silicone airway stents [16] and surgical tracheobronchoplasty [17]. Surgical central airway stabilization with posterior tracheobronchial splinting using a polypropylene mesh improves respiratory symptoms, health-related quality of life, and functional status in highly selected patients with severe symptomatic TBM [18].

The etiology of acquired TBM in many patients is not obvious. TBM is often seen in association with COPD in patients with a smoking history, thus leading to the inference that chronic inflammation and smoking are important contributing factors [19].

Nevertheless, little is known concerning the mechanism underlying the association between inflammation and TBM or EDAC. Loring et al. [6] speculated that repeated mechanical stress from coughing or the high expiratory pleural pressures during exercise in patients with airflow obstruction might cause stretching and degeneration of the posterior membranous portion of the trachea and main bronchi over a period of years.

At present very few data concerning TBM or EDAC epidemiology in asthma patients are available to our knowledge. Data from the present study, carried out in a consistent cohort of asthma patients of different severity and in whom the diagnosis of TBM o EDAC was confirmed via dynamic broncoscopy, ascertained that the prevalence of EDAC is directly related to asthma severity and is particularly high in severe asthma, while EDAC is much more frequent than TBM in asthmatic patients independently of asthma severity. This evidence supports the hypothesis that the chronic persistency of substantial airway inflammation within the airways can contribute to impair expiratory airflow just affecting intrinsically and persistently the structures of larger airways in a great proportion of cases.

Thyroid disease and its association with TBM was documented, but only when tracheal cartilaginous ring can be compromised by a tumour, or most commonly after thyroidectomy, especially in patients with cancer [19]. Furthermore, in our study, thyroid disorders proved highly concomitant in TBM and in EDAC, the latter condition being much more represented in females, independently of asthma severity.

## Conclusions

In conclusion, the presence of TBM or EDAC should be considered whenever bronchial asthma persists uncontrolled and unresponsive to an appropriate pharmacological treatment. Moreover, in severe asthma the presence of TBM or EDAC should be actively investigated, not only when uncontrolled, bearing in mind the range of non pharmacological treatment options that could be offered to these patients. Furthermore, if the occurrence of EDAC is highly probable in females with thyroid disturbances, it is as much appropriate that thyroid function be investigated in females with EDAC.

## Competing interests

The authors declare that they have no competing interests.
